# lncAKHE enhances cell growth and migration in hepatocellular carcinoma via activation of NOTCH2 signaling

**DOI:** 10.1038/s41419-018-0554-5

**Published:** 2018-04-30

**Authors:** Guanqun Huang, Hui Jiang, Ye Lin, Yanpeng Wu, Weilong Cai, Boyun Shi, Yuanwei Luo, Zhixiang Jian, Xinke Zhou

**Affiliations:** 1 Department of general surgery, the Fifth Affiliated Hospital of Guangzhou Medical University, Guangzhou, 510700 China; 2 Department of Abdominal Oncology, the Fifth Affiliated Hospital of Guangzhou Medical University, Guangzhou, 510700 China; 3 Department of General Surgery, Guangdong General Hospital, Guangdong Academy of Medical Sciences, Guangzhou, 510080 China

## Abstract

Hepatocellular carcinoma is the sixth most common cancer and gives rise to numerous deaths around the world every year. However, the molecular mechanism that controls hepatocarcinogenesis remains largely unknown. Here we found out an uncharacterized long noncoding RNA named lncAKHE. We found that lncAKHE was highly expressed in hepatocellular carcinoma tissues. lncAKHE depletion remarkably impaired the abilities of cell proliferation, migration, and invasion in hepatocellular carcinoma while promgoogoting cell apoptosis. Moreover, higher expression level of lncAKHE in hepatocellular carcinoma tissues was associated with more clinical severity and lower survival rates. Mechanistically, lncAKHE cooperated with YEATS4 to enhance the activation of NOTCH2 signaling which is usually abnormally upregulated in hepatocellular carcinoma. In conclusions, our study showed that lncAKHE may promote tumor progression in HCC and serve as a novel target for HCC treatment.

## Introduction

Hepatocellular carcinoma (HCC) is the sixth most common cancer and gives rise to numerous deaths around the world every year^1,2^. Currently, the curative therapies for HCC include radiofrequency ablation (RFA), transarterial chemoembolization (TACE) and intra-arterial radiotherapy (SIRT)^3,4^. For advanced HCC treatment, only surgery and local ablative therapies seem effective. Although some advance has been made on the methods for HCC treatment in recent years, the 5-year overall survival rate is still under 25%^5^. Therefore, developing new curative therapies for HCC is very necessary and urgent. HCC occurrence is a multistep and complex process associated with a variety of genetic mutations or epigenetic alterations^6–8^. Many efforts have been made to explore the potential mechanisms. However, the molecular mechanisms still remain elusive. Dysregulation of gene expression is proven to be essential for hepatocarcinogenesis^7^. So identifying genes that promote HCC development and progression is pivotal for revealing the mechanisms of hepatocarcinogenesis and developing effective therapies.

Long noncoding RNAs (lncRNAs) are a major class of transcripts of longer than 200 nucleotides (nt) and cannot code protein^9,10^. Recent researches showed that lncRNAs have important functions and were involved in diverse biological processes, such as development, immunity, and cancer^11–15^. LncRNAs perform functions in many kinds of ways, including regulating chromatin accessibility and gene expression^16^. For example, HOTAIR inhibits JAM2 transcription initiation by interacting with Ezh2 to reduce the chromatin accessibility^17^. Dysregulation of lncRNA expression may lead to many human diseases, especially tumor^18^. In most cancers, expression levels of many lncRNAs are abnormal. Therefore, exploring the functions of lncRNAs seems to be helpful for tumor treatment. However, the roles of large amounts of lncRNAs involved in tumorigenesis have not been defined. And the mechanisms by which lncRNAs function are still largely unknown.

In this study, we screened out a new lncRNA named as lncAKHE that was upregulated in HCC tissues compared to paired peritumor. We found that lncAKHE knockdown impaired cell proliferation and migration, and increased cell apoptosis. In mechanism, we found that lncAKHE interacted with YEATS4 to enhance NOTCH2 signaling activation which then promotes HCC progression. What’s more, patients with higher lncAKHE expression had lower survival rates. In summary, our study revealed a new mechanism of hepatocarcinogenesis and lncAKHE may serve as a potential target for HCC therapy.

## Results

### lncAKHE is upregulated in HCC tissues

**Fig. 1:**
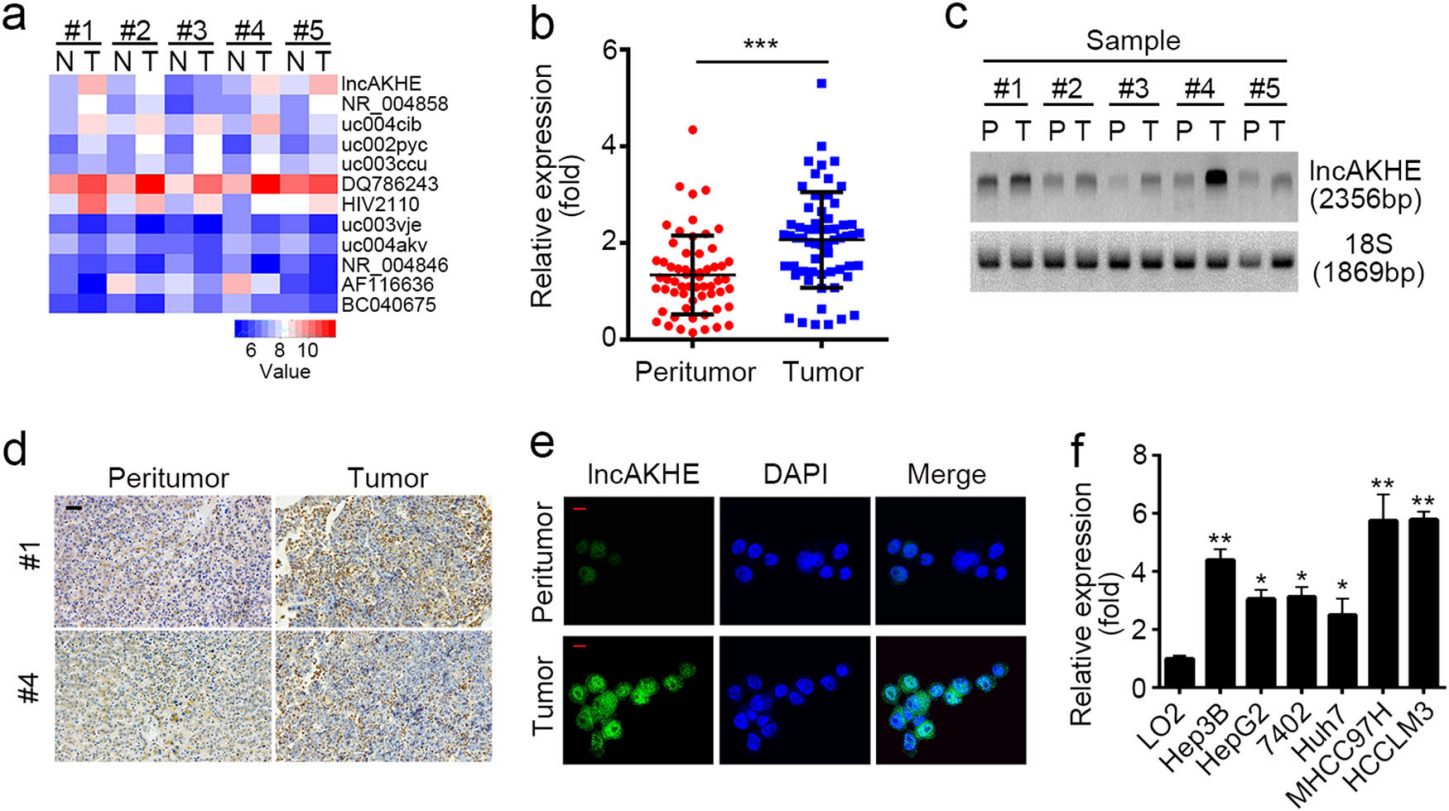
LncAKHE is upregulated in HCC tissues. **a** Microarray data in GSE27462 were analyzed. Differentially expressed lncRNAs were listed between HCC tissues and paired peritumor samples. **b** 60 pairs of peritumor and tumor samples were collected and total RNAs were extracted. Then the expression levels of lncAKHE were measured by RT-qPCR. **c** Expression levels of lncAKHE and 18S (loading control) were analyzed in peritumor and tumor tissues. Samples from #1 to #5 were chosen for Northern blot. lncAKHE and 18S probes were biotin-labeled. P peritumor, T tumor. **d** lncAKHE was expressed higher in HCC samples as shown by RNA hybridization in situ (ISH). lncAKHE probe was biotin-labeled. Scale bars = 100 μm. **e** The expression level of lncAKHE was checked in tumor cells by fluorescence in situ hybridization (RNA FISH). Scale bars = 10 μm. **f** Total RNAs were extracted from HCC cell lines. lncAKHE expression was examined by RT-qPCR. Data is from three independent experiments and expressed as mean ± SD. *p < 0.05, **p < 0.01, and ***p < 0.001

To explore how lncRNAs regulate HCC progression, we analyzed an online-available microarray dataset about lncRNAs expressed in HCC (GSE27462)^19^. We screened out upregulated or downregulated lncRNAs in all five HCC samples compared to paired peritumor tissues (Fig. 1a). Then we focused on a most upregulated and uncharacteristic lncRNA (AK056594 located on chr8: 67,245,089 ~ 67,247,203). We named it as lncAKHE for abbreviation of AK056594 and highly expression in HCC. Then we analyzed the expression of lncAKHE in 60 pairs of HCC and peritumor samples. We found that lncAKHE was upregulated in HCC tissues (Fig. 1b). To further confirm it, we chose five pairs of HCC samples for Northern bot and got the same result (Fig. 1c). What’s more, we used biotin-labeled lncAKHE specific probes for in situ hybridation (ISH) and RNA Fluorescence in situ hybridation (RNA FISH), and found that lncAKHE was highly expressed in HCC samples (Fig. 1d, e). Besides, according to qPCR, lncAKHE was also expressed higher in HCC cell lines including Hep3B, HepG2, 7402, Huh7, MHCC97H, and HCCLM3 cells compared to human normal hepatocyte LO2 (Fig. 1f). In sum, lncAKHE was upregulated in HCC tissues.

### lncAKHE is positively correlated with HCC severity

**Fig. 2:**
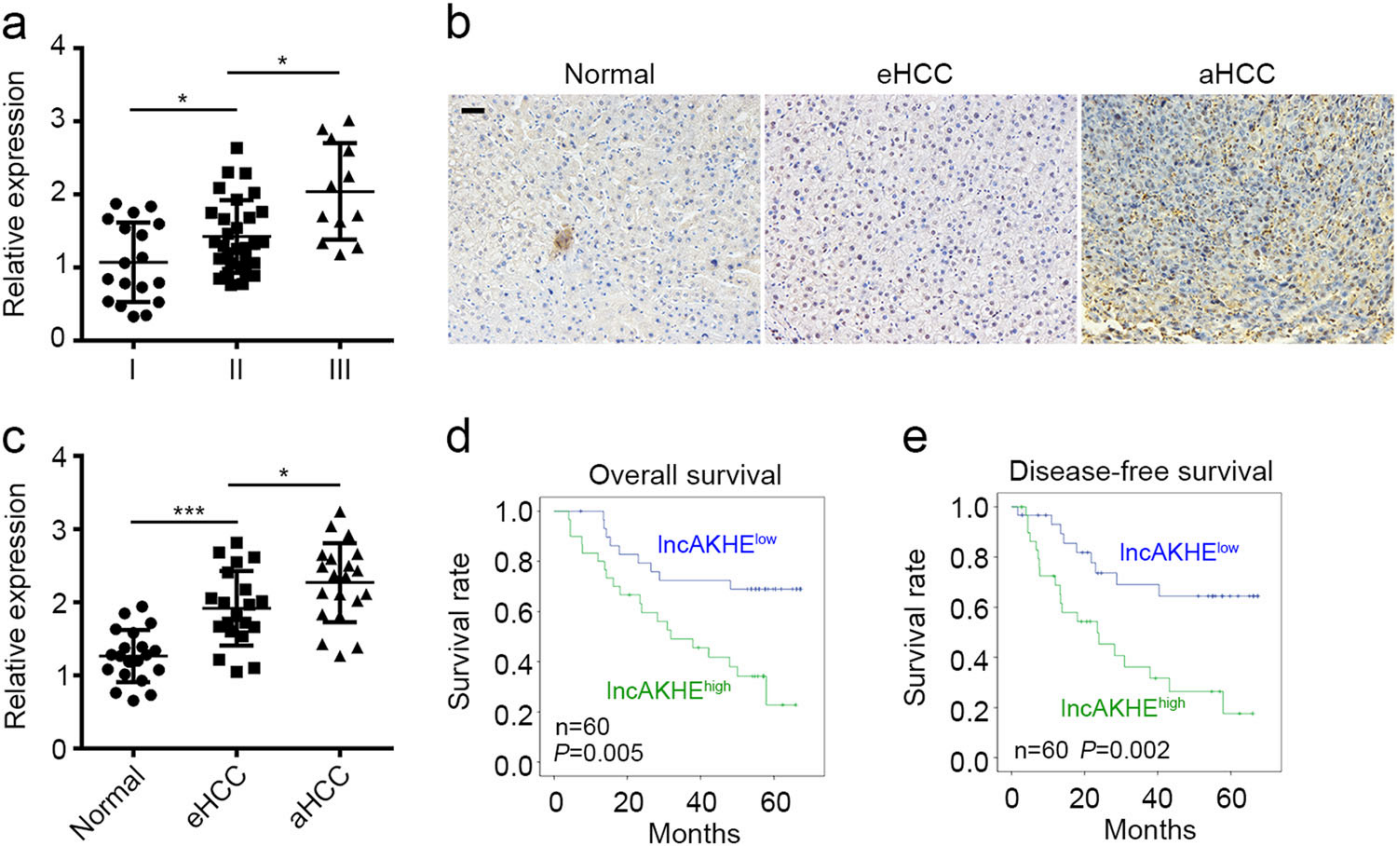
LncAKHE is positively correlated with HCC severity. **a** HCC samples were divided into three groups based on the clinical stage (18 samples in stage I, 30 samples in Stage II, and 12 samples in stage III). Then the expression of lncAKHE was analyzed by RT-qPCR. **b** lncAKHE expression was checked by ISH in non-tumor, early HCC (eHCC) and advanced HCC (aHCC) tissues. Scale bar = 100 μm. **c** Total RNAs were extracted from non-tumor, eHCC, and aHCC samples. lncAKHE expression was analyzed by RT-qPCR. **d**, **e** The survival rates in patients with higher lncAKHE expression were lower. Sixty HCC samples were divided into two groups based on the levels of lncAKHE expression. Then Kaplan–Meier survival analyses were conducted. Data is from three independent experiments and expressed as mean ± SD. *p < 0.05, **p < 0.01, and ***p < 0.001

We further divided 60 pairs of HCC samples into three groups according to AJCC TNM classification. We found that lncAKHE was expressed higher in samples of stage II and Stage III (Fig. 2a). Besides, more lncAKHE was expressed in early HCC (eHCC) and advanced (aHCC) samples than normal liver tissues (Fig. 2b, c). Then we grouped these samples into high and low expression subsets according to lncAKHE expression. Kaplan–Meier survival analysis was performed. We found that HCC patients with higher lncAKHE expression possessed poorer survival rates (Fig. 2d, e). Collectively, lncAKHE is positively correlated with HCC severity.

### lncAKHE knockdown inhibits tumor cell proliferation and promotes apoptosis

**Fig. 3:**
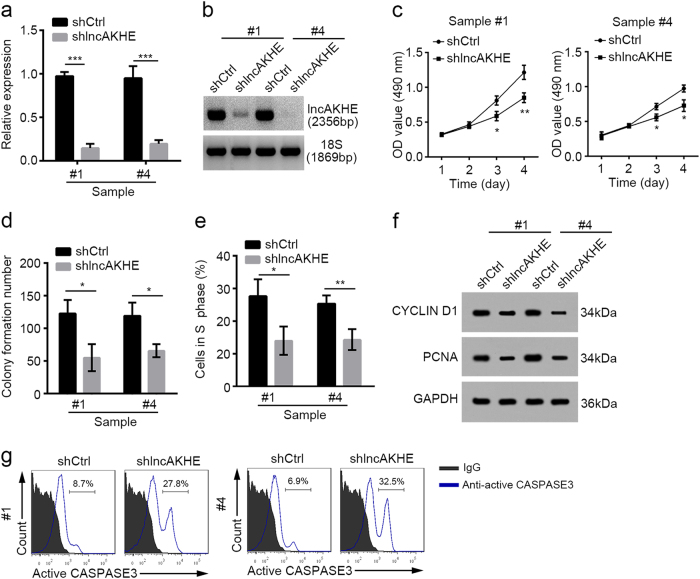
LncAKHE knockdown inhibits tumor cell proliferation and promotes apoptosis. **a**, **b** Tumor cells from sample #1 and #4 were cultured and infected with pSICOR-GFP-shlncAKHE lentivirus. Then GFP^+^ cells were isolated by FACS. The efficiency of lncAKHE silence was analyzed by RT-qPCR (**a**) and Northern blot (**b**). Cells infected with scramble shRNA were chosen for control. **c,** **d** The proliferative capacity of shCtrl or shlncAKHE sample cells were measured by MTS assays (**c**) and colony formation assays (**d**). **e** The percentage of shCtrl or shlncAKHE sample cells in S phase was examined by FACS. **f** The protein levels of CYCLIN D1 and PCNA were decreased in shlncAKHE sample cells. GAPDH was chosen as loading control. **g** More active CASPASE3^+^ cells appeared after lncAKHE depletion as shown by FACS analysis. Data is from three independent experiments and expressed as mean ± SD. *p < 0.05 and **p < 0.01

We then explored the effect of lncAKHE on tumor cells. We collected HCC samples and cultured them. By infecting tumor cells from sample #1 and #4 with virus containing shlncAKHE, we successfully knocked down lncAKHE as shown by qPCR and Northern blot (Fig. 3a, b). According to MTS assays, we found that lncAKHE-depleted tumor cells showed impaired proliferation ability (Fig. 3c). What’s more, lncAKHE-silenced cells formed less clones (Fig. 3d) and less cells entered into S phase (Fig. 3e). Consistently, lncAKHE knockdown decreased the expression of proliferation-related proteins including CYCLIN D1 and PCNA (Fig. 3f). On the other hand, lncAKHE depletion also promoted cell apoptosis (Fig. 3g). In a word, lncAKHE knockdown seriously inhibited cell proliferation and promoted apoptosis.

### lncAKHE knockdown impairs tumor cell migration and invasion

**Fig. 4:**
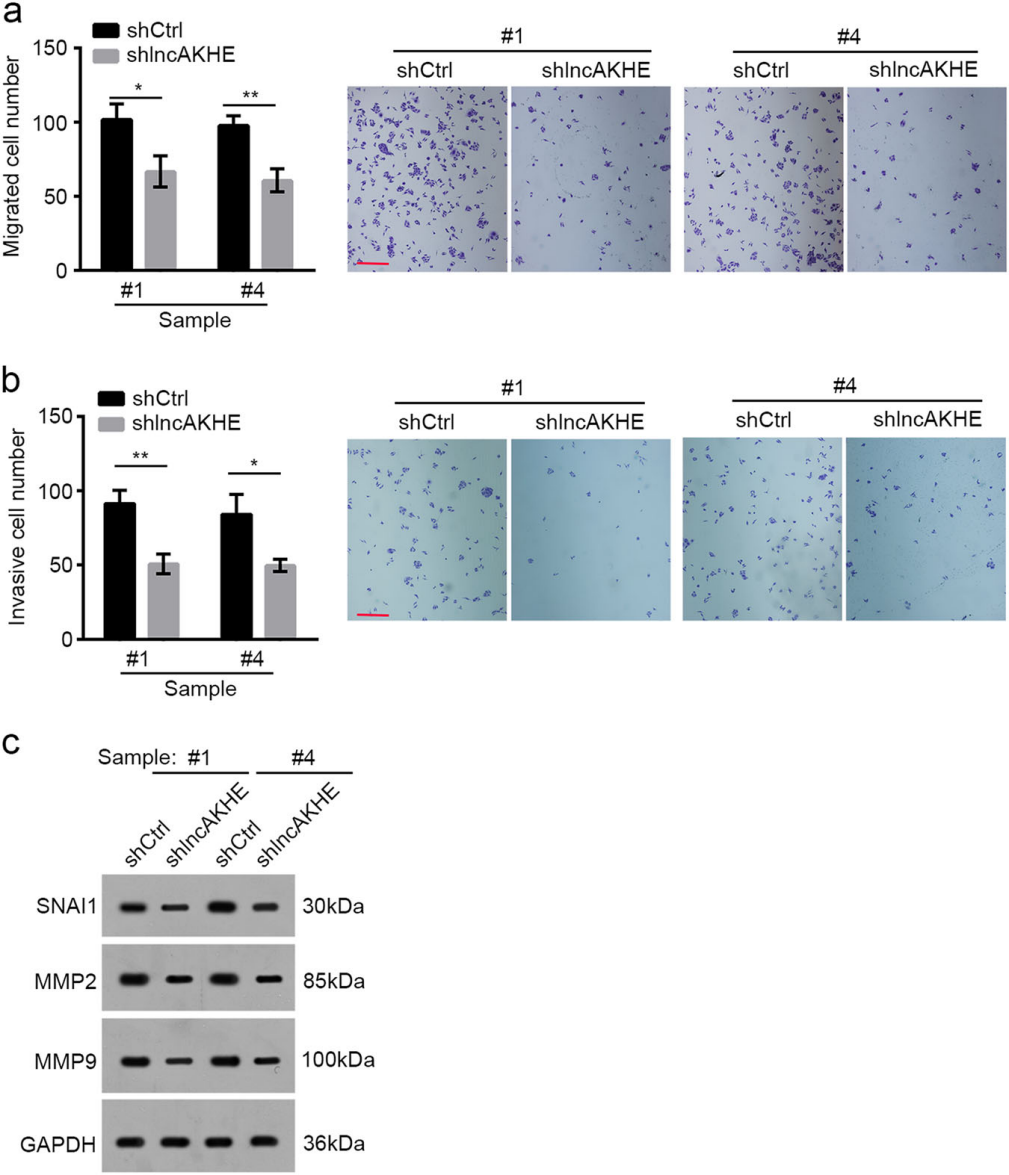
LncAKHE knockdown impairs tumor cell migration and invasion. **a** lncAKHE knockdown decreased the migration potential of HCC sample cells in a Transwell migration assay. Scale bar = 50 μm. **b** lncAKHE knockdown significantly inhibited the invasion capacity of HCC sample cells in a Matrigel invasion assay. Scale bar = 50 μm. **c** The protein levels of SNAI1, MMP2, and MMP9 were decreased in lncAKHE-silenced tumor cells. GAPDH was chosen as loading control. Data is from three independent experiments and expressed as mean ± SD. *p < 0.05 and **p < 0.01

We found a significant correlation between lncAKHE expression and TNM stages. We subsequently conducted cell migration and invasion assays with lncAKHE-silenced tumor cells to determine the potential relationship between lncAKHE expression and metastasis. We found that lncAKHE knockdown remarkably reduced the migration and invasion potential of tumor cells (Fig. 4a, b). Consistently, lncAKHE knockdown also decreased the expression levels of SNAI1, MMP2, and MMP9 (Fig. 4c). Summarily, our study indicated that lncAKHE may promote cell metastasis in HCC patients.

### lncAKHE promotes tumor propagation in vivo

**Fig. 5:**
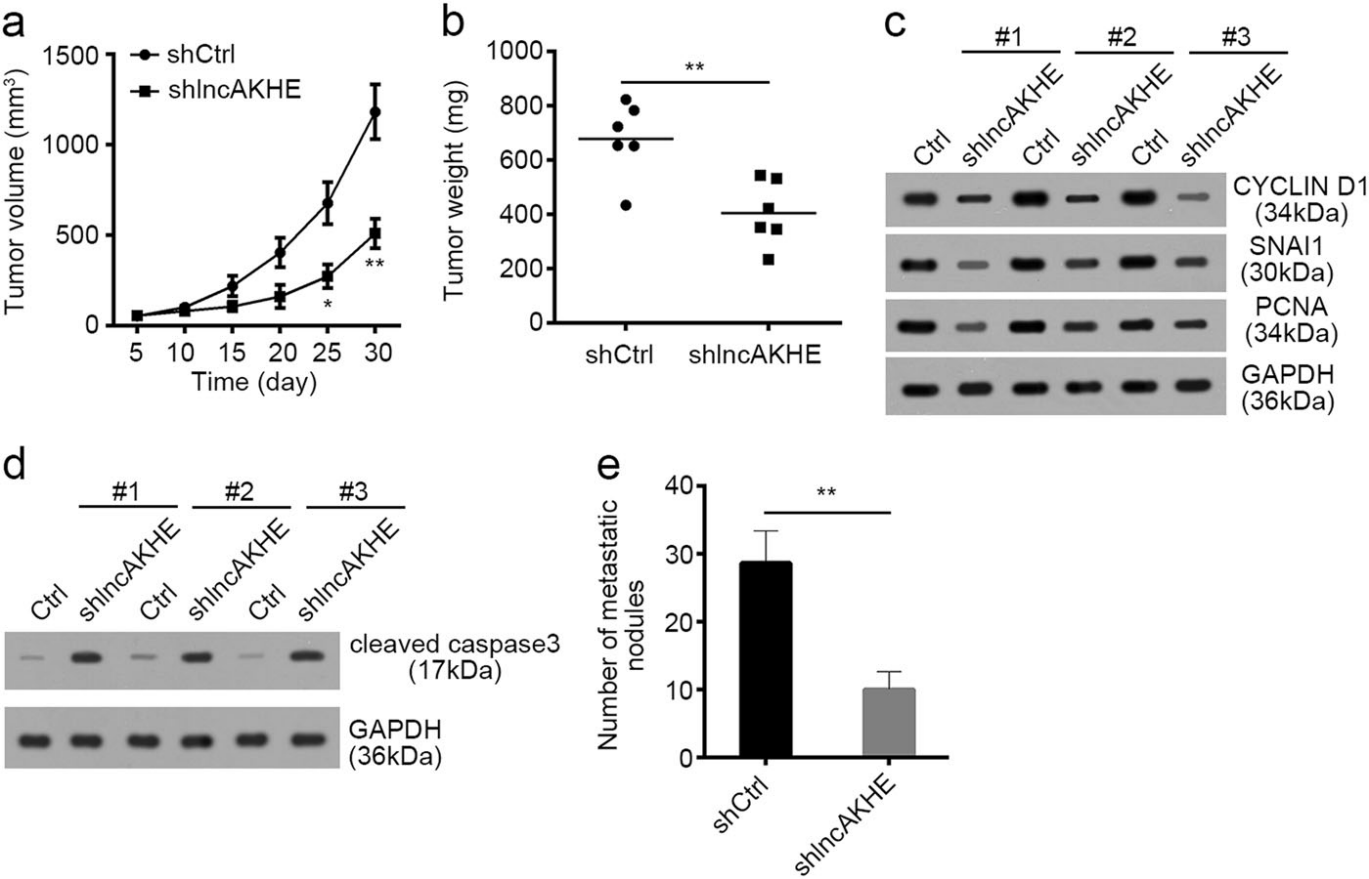
LncAKHE promotes tumor propagation in vivo. **a** 2 × 10^6^ lncAKHE-depleted or control cells were injected into nude mice. The volumes of tumors were calculated at indicative time points. LncAKHE depletion significantly impaired the rate of tumor growth in vivo. **b** 2 × 10^6^ lncAKHE-depleted or control cells were injected into nude mice. Tumor weights were measured at 30 days post injection. The bigger tumors were originated from shCtrl tumor cells. **c** The protein levels of CYCLIN D1, SNAI1, and PCNA were checked in the formed tumors of (**b**). ShlncAKHE cell-derived tumors have impaired proliferation and migration abilities. **d** The protein level of cleaved caspase3 was measured in formed tumor tissues. **e** Quantification of the number of metastatic nodules in the lung. *n* = 6 in each group. Data is from three independent experiments and expressed as mean ± SD. **p < 0.01

To further explore the role of lncAKHE in vivo, we performed a xenograft nude mouse experiment via subcutaneously injecting shCtrl or shlncAKHE HCC sample cells. We found that lncAKHE knockdown significantly delayed the tumor growth (Fig. 5a). And the tumor weights were also decreased on 30th day post injection after lncAKHE depletion (Fig. 5b). Furthermore, the proliferation and invasion abilities of formed tumors derived from lncAKHE-silenced tumor cells were also impaired (Fig. 5c). Consistently, knockdown of lncAKHE also led to increased apoptosis in formed tumor cells, as higher levels of cleaved caspase3 were observed in shlncAKHE tumor cells than in control group (Fig. 5d). To explore how lncAKHE regulates tumor metastasis in vivo, we intravenously injected 5 × 10^5^ WT or shlncAKHE sample cells into lateral tail veins of recipient mice. The mice were sacrificed 4 weeks later, and the lung metastases were analyzed. As shown, lncAKHE knockdown decreased the nodules in the lung (Fig. 5e).

### LncAKHE interacts with YEATS4

**Fig. 6:**
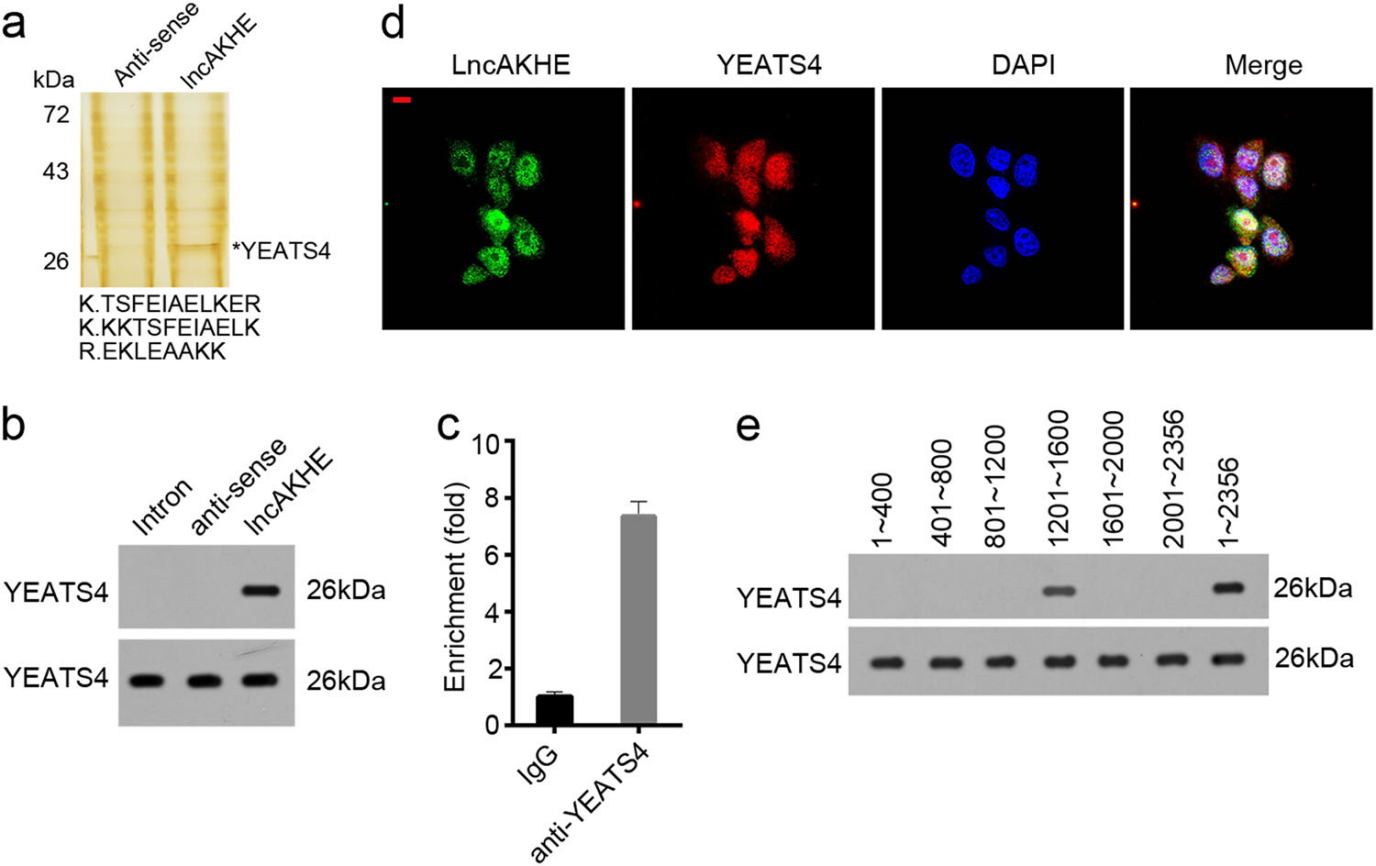
LncAKHE interacts with YEATS4. **a** Biotin labeled lncAKHE and anti-sense controls were used for RNA pulldown assays. Then SDS-PAGE, silver staining and mass spectrometry were conducted sequentially. YEATS4 was identified as a potential interactive protein of lncAKHE. **b** Biotin labeled lncAKHE and control sequences were added into sample lysates and RNA pulldown assays were conducted. **c** Anti-YEATS4 was added into sample lysates and RNA IP assay was performed. **d** LncAKHE was co-localized with YEATS4 in the nucleus of sample cells. Green lncAKHE, Red YEATS4, Blue DAPI. Scale bar = 10 μm. **e** Domain mapping showed that region 1201 ~ 1600 in lncAKHE was essential for its association with YEATS4. Data is from three independent experiments and expressed as mean ± SD. **p < 0.01

Previous studies showed that lncRNA can associates with proteins to regulate gene expression^14,20^. To screen out potential lncAKHE-interactive proteins, we performed RNA pulldown and silver staining with HCC sample cell lysates, followed by identification with mass spectrometry (MS) (Fig. 6a). YEATS4, a transcription activator, was identified as a potential interactive protein. We validated their interaction by RNA pulldown and RNA immune-precipitation (RNA IP) assays (Fig. 6b, c). Immunofluorescence also showed that lncAKHE co-localized with YEATS4 in HCC sample cells (Fig. 6d). Domain mapping also indicated that the region of nt: 1201 ~ 1600 in lncAKHE is indispensible for its interaction with YEATS4 (Fig. 6e).

### LncAKHE activates NOTCH2 pathway

**Fig. 7:**
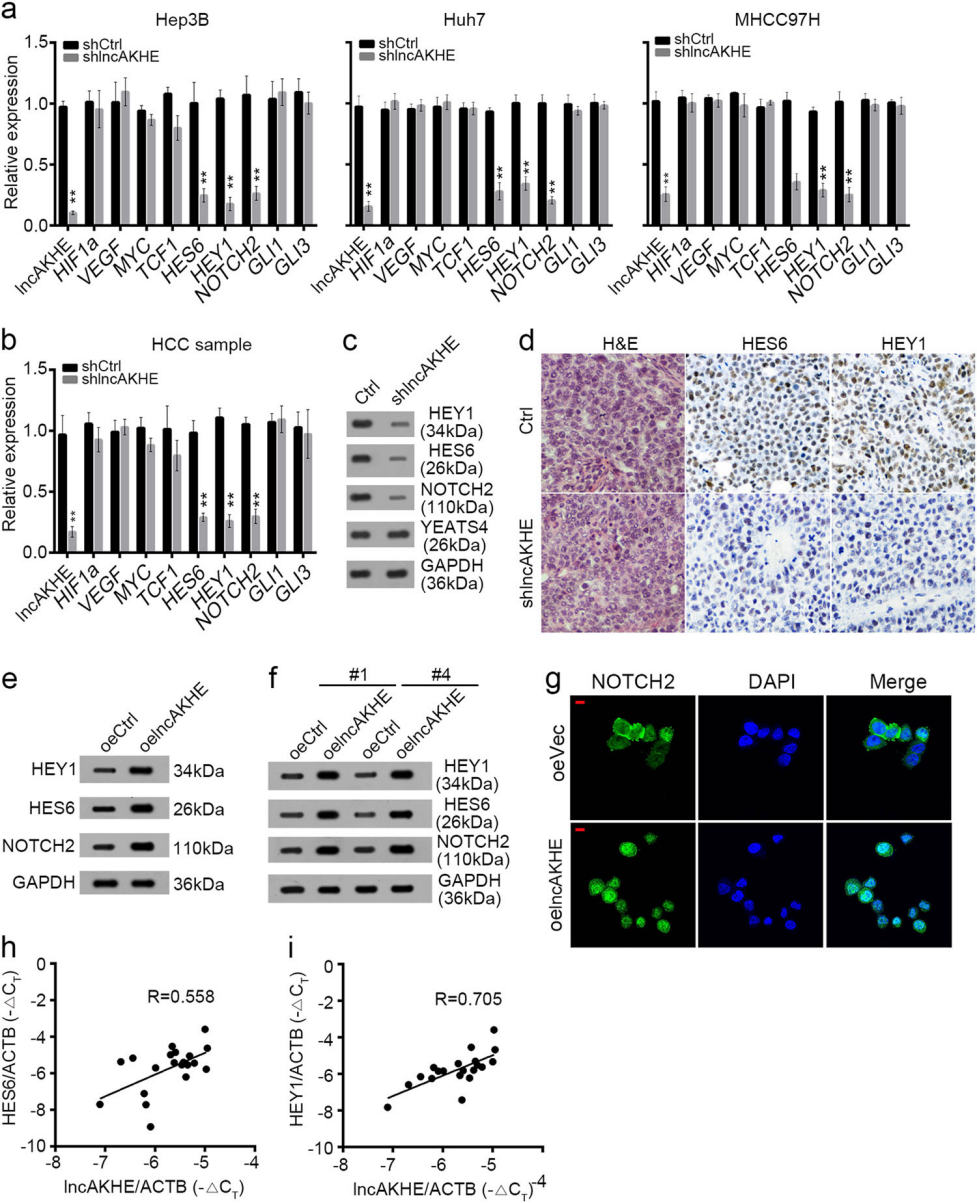
LncAKHE activates NOTCH2 pathway. **a** LncAKHE was knocked down in Hep3B, Huh7, and MHCC97H cells. Then the target gene expression of NF-κB, Wnt/β-catenin, NOTCH2, and Hedgehog signaling was examined by qPCR. LncAKHE depletion inhibited NOTCH2 signaling. **b** NOTCH2 signaling was also impaired in lncAKHE-silenced HCC cells.**c** Protein levels of YEATS4, NOTCH2, HES6, and HEY1 in formed tumor tissues of the xenograft experiment. **d** Expression levels of HES6 and HEY1 in formed tumor tissues of the xenograft experiment were measured by IHC staining. **e** and **f** The protein levels of NOTCH2, HEY1, and HES6 were increased in lncAKHE overexpressing Hep3B (**e**) and HCC sample (**f**) cells. GAPDH was chosen as loading control. **g** LncAKHE overexpression promotes NOTCH2 entry into nucleus in HCC sample cells. Scale bar = 10 μm. **h** and **i** The expression levels of HES6 (**h**) or HEY1 (**i**) were positively correlated with that of lncAKHE in HCC samples. Data is from three independent experiments and expressed as mean ± SD. **p < 0.01

Accumulating evidences indicated that NF-κB, Wnt/β-catenin, NOTCH, and Hedgehog signaling pathways play essential roles in the development and progression of HCC^21,22^. To explore the molecular mechanism of lncAKHE function, we analyzed the effect of lncAKHE on NF-κB, Wnt/β-catenin, NOTCH, and Hedgehog signalings. We explored the expression of their target genes (*HIF1A* and *VEGFA* for NF-κB, *MYC* and *TCF1* for Wnt/β-catenin, *NOTCH2*, *HES6* and *HEY1* for NOTCH, *GLI1* and *GLI3* for Hedgehog) by qPCR in Hep3B, Huh7, and MHCC97H cells, and found that the NOTCH2 signaling was downregulated after lncAKHE knockdown in these three cell lines (Fig. 7a). The same result was observed in HCC sample cells (Fig. 7b). Furthermore, we checked the protein levels of NOTCH2, YEATS4, HEY1, and HES6 in tumor tissues of the xenograft experiment. We found that the expression levels of NOTCH2, HEY1, and HES6 were lower in lncAKHE-silenced HCC cell-derived tumor tissues than in control tumor tissues, whereas YEATS4 expression was not affected (Fig. 7c). IHC staining also showed that lncAKHE depletion led to reduced expression of HES6 and HEY1 in lncAKHE-silenced tumor tissues (Fig. 7d).

Then we overexpressed lncAKHE in Hep3B and HCC sample cells. We found that lncAKHE overexpression activated NOTCH2 signaling (Fig. 7e, f). LncAKHE overexpression promoted NOTCH2, HEY1, and HES6 expression in Hep3B and HCC sample cells (Fig. 7e, f). Consistently, overexpressing lncAKHE promoted NOTCH2 expression and entry into nucleus (Fig. 7g). Then we analyzed the relationship between NOTCH2 signaling activation and lncAKHE expression in HCC samples and found that lncAKHE expression was positively correlated with that of HES6 or HEY1 (Fig. 7h, i).

### lncAKHE recruits YEATS4 to initiate NOTCH2 expression and activate NOTCH2 signaling

**Fig. 8:**
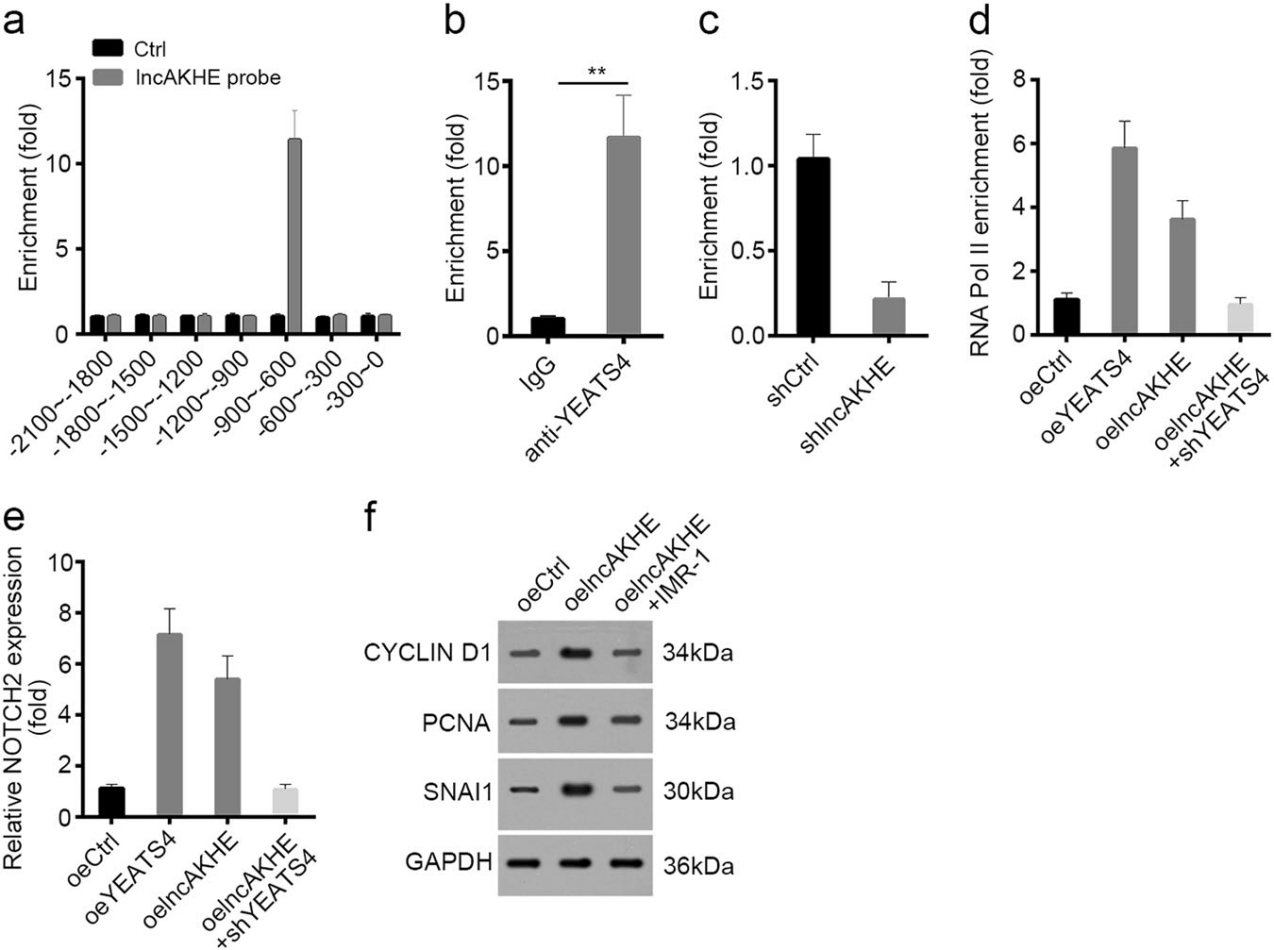
LncAKHE recruits YEATS4 to initiate NOTCH2 expression and activate NOTCH2 signaling. **a** LncAKHE enriched on NOTCH2 promoter by CHIRP assay. LncAKHE probes were biotin-labeled. Genomes from HCC sample lysates were sonicated into fragments of ~ 500 bp and incubated with lncAKHE probes. **b** YEATS4 was enriched on NOTCH2 promoter as shown by ChIP assays. **c** LncAKHE knockdown impaired the enrichment of YEATS4 on NOTCH2 promoter. **d** Overexpressing YEATS4 or lncAKHE promoted the enrichment of RNA Pol II on NOTCH2 promoter. **e** Overexpressing YEATS4 or lncAKHE promoted the expression of NOTCH2. **f** LncAKHE overexpression promoted cell proliferation and migration while inhibition of NOTCH signaling by addition of IMR-1 reversed it. Data is from three independent experiments and expressed as mean ± SD. **p < 0.01

To further explore how lncAKHE activates NOTCH2 signaling, we conducted chromatin isolation by RNA purification (CHIRP) assays to define whether lncAKHE directly regulates NOTCH2 expression. We found that lncAKHE enriched on NOTCH2 promoter (region of −900 ~ −600 bp from TSS) (Fig. 8a). We above demonstrated that lncAKHE associated with YEATS4 in HCC. To further explore whether YEATS4 also regulates NOTCH2 expression, we performed ChIP assays and found that YEATS4 bound to the same region of NOTCH2 promoter (−900 ~ −600 bp from TSS) (Fig. 8b). Surprisingly, lncAKHE knockdown seriously impaired the enrichment of YEATS4 on NOTCH2 promoter (Fig. 8c), which indicated that lncAKHE and YEATS4 may promote NOTCH2 transcription coordinately. Consistently, overexpressing YEATS4 or lncAKHE really promoted the enrichment of RNA Pol II on NOTCH2 promoter and NOTCH2 mRNA level (Fig. 8d, e). Nevertheless, YEATS4 knockdown abrogated the effect of lncAKHE on NOTCH2 expression (Fig. 8d, e). Above results showed that lncAKHE recruited YEATS4 to NOTCH2 promoter, initiated NOTCH2 transcription and then activated NOTCH2 pathway.

To determine whether NOTCH2 pathway is dispensable for the function of lncAKHE in HCC, we inhibited NOTCH2 pathway in lncAKHE-overexpressing cells. As shown, overexpressing lncAKHE promoted the expression of proliferaton- and metastasis-related proteins while inhibition of NOTCH2 pathway with IMR-1 abrogated it (Fig. 8f). Summarily, our research showed that lncAKHE recruited YEATS to initiate NOTCH2 expression and activate NOTCH2 signaling pathway. Then abnormal NOTCH2 signaling activation is responsible for lncAKHE-mediated HCC cell proliferation, migration, and invasion at least partially.

## Discussion

Hepatocellular carcinoma (HCC) is the sixth most common cancer and gives rise to numerous deaths around the world every year^23,24^. Although some advance has been made in the methods for HCC treatment recent years, the 5-year overall survival rate is still very low. Many studies have shown that some genetic mutations or abnormal activation of signaling pathways lead to hepatocarcinogenesis. For example, Pei et al. reported that FGF8 promotes HCC growth via activation of EGFR^25^. Activation of Wnt/β-catenin leads to cell proliferation in HCC^26^. However, the molecular mechanism of hepatocarcinogenesis still remains to be explored. In our study, we screened out a new lncRNA named lncAKHE that was highly expressed in HCC tissues. LncAKHE knockdown significantly inhibited cell proliferation and invasion in HCC. Moreover, lncAKHE-silenced tumor cells showed reduced growth ability in vivo. Therefore, lncAKHE has great promise as a novel diagnostic and prognostic marker.

Accumulated evidences showed that there was a closely relationship between lncRNAs expression and tumorigenesis, metastasis or prognosis^27–30^. LncRNAs can serve as tumor suppressors or oncogenes by regulating gene expression or pivotal signaling pathway, such as Wnt/β-catenin and NOTCH signaling^31,32^. We found that lncAKHE acted as an oncogene by activating NOTCH2 signaling. Through knocking down lncAKHE, we found that NOTCH signaling was downregulated. The protein levels of HEY1 and HES6, the target genes of NOTCH signaling, were greatly increased by lncAKHE overexpression. What is more, lncAHKE overexpression promoted NOTCH2 expression and entry into nucleus by IF staining. In the presence of NOTCH signaling inhibitor, lncAKHE overexpression cannot promote cell proliferation and migration in HCC. So our study showed that the function of lncAHKE in HCC was dependent on NOTCH2 signaling. Our study revealed a novel lncRNA in HCC and demonstrated its functions, which provides a new insight on the mechanism of HCC progression. However, we showed there were differential expression levels of lncAKHE in HCC patients, so the mechanism regulating lncAKHE expression in HCC remains to be further determined. Although we showed lncAKHE promoted HCC cell proliferation, migration and invasion in vitro and in vivo, whether lncAKHE promotes hepatocarcinogenesis in physical condition needs further investigation. A lncAKHE knockout mouse model may better explain the function of lncAKHE in hepatocarcinogenesis. Furthermore, NOTCH pathway has been shown to regulate the self-renewal of HCC stem cells^21^. Whether lncAKHE/NOTCH2 axis exerts a role on HCC stem cells also requires to be explored.

Recently, increasing evidences demonstrated that lncRNA can act as a scaffold to recruit proteins for regulation of gene expression^9,33^. To define the molecular mechanism of lncAKHE promoting proliferation and migration in HCC by NOTCH2 signaling, we performed RNA pulldown assays to search potential interactive proteins. We screened out YEATS4 as a candidate. Firstly, we validated the interaction of lncAKHE and YEATS4. Then we showed that lncAKHE and YEATS4 all bound to NOTCH2 promoter. Overexpressing lncAKHE or YEATS4 promoted RNA Pol II enrichment on NOTCH2 promoter, which suggested that lncAKHE and YEATS4 directly promoted NOTCH2 expression. Although we demonstrated lncAKHE mainly enhanced NOTCH2 expression in a direct manner, whether lncAKHE also upregulated NOTCH2 pathway activation indirectly has not been excluded. YEATS4, a transcription activator, has been reported to participate in many tumors. For example, downregulation of YEATS4 promoted cell apoptosis in colorectal cancer cells^34^. YEATS4 promoted cell proliferation via activating the Wnt/β-catenin signaling in gastric cancer^35^. YEATS4 acts as an oncogene in non-small cell lung cancer^36^. However, the function of YEATS4 in HCC has not been defined. By analysis of YEATS4 expression, we found that YEATS4 was also highly expressed in HCC. According to our all results, YEATS4 also served as an oncogene in HCC. However, as a chromatin remodeling protein, YEATS4 will regulate a large range of gene expression via various mechanisms. Therefore, the physiological functions of YEATS4 in HCC may be not completely the same as lncAKHE, which need more investigation. And the mechanism that YEATS4 exerts functions in lncAKHE-independent manner in HCC remains to be defined.

## Materials and methods

### Patient samples

For this study, we collected 60 HCC samples from the Fifth Affiliated Hospital of Guangzhou Medical University. We listed the samples’ clinical characteristics in Supplementary Table 1. For tumor cell culture, we cut HCC tissues into pieces and digested using collagenase IV for 50 min at 37 °C. We obtained consents that approve usage of these HCC tissues in this study from all patients. All the experiments were approved by the Fifth Affiliated Hospital of Guangzhou Medical University. The study protocol was approved by the Fifth Affiliated Hospital of Guangzhou Medical University.

### Cell lines

The normal liver cell line LO2 and The HCC cell lines (Hep3B, 7402, Huh7, and HepG2) were obtained from ATCC. All cells were cultured in RPMI 1640 medium (Invitrogen, Shanghai, China) supplemented with 10% fetal bovine serum (FBS; Invitrogen) and 100 U/ml penicillin plus 100 μg/ml streptomycin.

### ShRNA-mediated interference

shlncAKHE and shYEATS4 were designed and obtained from Invitrogen. The shRNA sequences were as follows: lncAKHE: 5ʹ-TCCTGATTCTCTCCCTCCTCC-3ʹ, YEATS4: 5ʹ-AACAGTATATGTGAAACCATAT-3ʹ. shRNAs were constructed into pSICOR-GFP plasmid. To produce lentivirus, pSICOR-GFP-shlncAKHE was transfected into 293T cells along with VSVG, RRE, and REV plasmids through a Lipofectamine 3000 kit (Invitrogen). 24 h later, the medium was collected and added with PEG5000. Then we collected virus by centrifugation, followed by infection into tumor cells. GFP^+^ cells were purified by FACS and the depletion of lncAKHE or YEATS4 was validated by qPCR or WB. Control tumor cells were infected with virus containing pSICOR-GFP-scramble.

### Antibodies

Anti-GAPDH (5174), anti-CYCLIN D1 (2978), anti-SNAI1 (3879), anti-MMP2 (87809), anti-MMP9 (13667), anti-NOTCH2 (5732), and anti-PCNA (13110) were from Cell Signaling Technology. Anti-YEATS4 (sc-81278), anti-HEY1 (sc-134362) and anti-HES6 (sc-133196) were from Santa Cruz. Anti-active CASPASE (3550914) was from BD Bioscience.

### Xenograft assays

Ten male BALB/c nude mice (6 weeks old) were purchased from HFK Biosciences. Each mouse was subcutaneously injected with 2 × 10^6^ shCtrl tumor cells on the left flank region and shlncAKHE tumor cells on the right flank region. The tumor volumes were measured at indicative time points. After 30 days, the weights of tumors were analyzed. Animal experiments were performed in accordance with relevant guidelines and regulations of the Institutional Animal Care and Use Committees at the Fifth Affiliated Hospital of Guangzhou Medical University, and protocols were approved by the Institutional Animal Care and Use Committees at the Fifth Affiliated Hospital of Guangzhou Medical University.

### Cell proliferation assay

An amount of 1 × 10^3^ cells were seeded in 96-well plates and cultured. Cell proliferation was measured by an MTS cell proliferation assay every other day. A volume of 20 μL MTS was added and incubated for 2 h at 37 °C. Then the absorbance was analyzed at 490 nm.

### Migration and invasion assays

Cell migration and invasion assays have been described before^37^. In brief, 2 × 10^3^ cells/ well were seeded into the upper chamber of 24-well chambers and cultured in serum-free medium. The lower chamber was added medium with 10% FBS. 48 h later, migrating cells were fixed and stained with 0.5% crystal violet. Then three randomly fields were counted with a microscope. For invasion assay, the seeded cells were pre-coated with 500 ng/ml Matrigel (BD). Other steps were the same as migration assay.

### Real-time quantitative PCR

Total RNAs were isolated using TRIzol reagent from HCC samples according to the manufacturer’s protocol. cNDA were synthesized and used for analysis of mRNA transcripts using ABI 7300 qPCR system. Relative expressions were calculated and normalized to endogenous *ACTB*. Specific primer sequences were listed in Supplementary Table 2.

### Chromatin immunoprecipitation (ChIP) assay

ChIP assay were performed as described before^38^. In brief, HCC cells were fixed with 1% formaldehyde at 37 °C for 10 min. Then cells were lysed and genomes were sonicated into ~ 500 bp fragments. Specific antibodies were added into lysates and incubated overnight at 4 °C. Then DNA fragments were enriched by Protein A Agarose/Salmon Sperm DNA (50% Slurry) beads and purified, followed by qPCR analysis. Specific primer sequences were listed in Supplementary Table 3.

### LncRNA in situ hybridization

The expression of lncAKHE in HCC tissues were measured with biotin-labeled lncAKHE probes. Paraffinized sections were deparaffinized with xylene and 100% ethanol. Then sections were incubated with biotin-labeled probes for 18 h at 40 °C. DAB substrate was used for colorimetric detection of lncAKHE. Finally, the sections were co-stained with hematoxylin, followed by dehydration in graded alcohols and xylene. Biotin-conjugated probes were purchased from Invitrogen. lncAKHE probe sequences as follows: #1: 5′-TTGGGTTAGGCAAACACTGT-3′; #2: 5′-ACAACAGATTGATAGTCCAT-3′; #3: 5′-ACAATAGCACCCCAATAAGA-3′.

### Northern blot

RNAs were isolated from HCC samples and cell lines using TRIZOL (Invitrogen). lncAKHE and 18S probes for Northern blot were achieved by Biotin RNA labeling mix (Roche). The RNA samples were separated by electrophoresis and transferred to NC membrane. Then the membranes were incubated with hydration buffer containing probes. Finally, RNA signal was detected with Chemiluminescent Nucleic Acid Detection Module (Thermo Scientific).

### Pulldown and mass spectrometry

HCC lysates were incubated with biotin-labeled lncAKHE at 4 °C overnight. Then beads were added for incubation for another 4 h. Finally, beads were collected and washed. Then SDS-PAGE was performed, followed by silver staining. Differential bands that appeared in lncAKHE lane were cut off for mass spectrometry (LTQ Orbitrap XL). Biotin-labeled lncAKHE was achieved by a T7 transcription in vitro kit (Roche).

### Statistical analysis

All statistical analyses were performed using the Statistical Package for the Social Sciences version 20.0 software (SPSS Inc., Chicago, IL, USA). Survival curves were calculated using the Kaplan-Meier method and were analyzed using the log-rank test. For comparisons, one-way analyses of variance, Fisher’s exact tests, chi-squared tests, and two-tailed Student’s *t*-tests were performed, as appropriate. *P* < 0.05 was considered statistically significant.

## Electronic supplementary material

Supplementary data
