# Supplementary material for: lncAKHE enhances cell growth and migration in hepatocellular carcinoma via activation of NOTCH2 signaling
Source: Cell Death Dis. 2018 Apr 30;9(5):487. doi: 10.1038/s41419-018-0554-5 (PMC5924759; doi:10.1038/s41419-018-0554-5)
Supplement: Supplementary file 1 — Supplementary data [file 41419_2018_554_MOESM1_ESM.docx]

**Supplementary Table 1. Clinical Characteristics and Outcome of 60 HCC Patients According to lncAKHE Expression Levels**

| Feature | lncAKHE  Low | High* | Chi-square | P value |
| --- | --- | --- | --- | --- |
| All cases | 30 | 30 |  |  |
| Age |  |  | 0.131 | 0.718 |
| <60 | 25 | 26 |  |  |
| ≥60 | 5 | 4 |  |  |
| Gender |  |  | 1.176 | 0.278 |
| Male | 24 | 27 |  |  |
| Female | 6 | 3 |  |  |
| AFP |  |  | 0.268 | 0.518 |
| <400 | 13 | 15 |  |  |
| ≥400 | 17 | 15 |  |  |
| Size (cm) |  |  | 0.884 | 0.347 |
| <3 | 8 | 5 |  |  |
| ≥3 | 22 | 25 |  |  |
| BCLC |  |  | 0.300 | 0.584 |
| A | 11 | 9 |  |  |
| B or C | 19 | 21 |  |  |
| TNM |  |  | 6.667 | 0.010 |
| I/II | 28 | 20 |  |  |
| III/IV | 2 | 10 |  |  |

*The median expression level was used as the cutoff.

For analysis of correlation between lncAKHE levels and clinical features, Pearson’s chi-square tests were used. Results were considered statistically significant at *P*<0 .05.

Abbreviations: AFP, alpha-fetoprotein; BCLC, Barcelona Clinic liver cancer staging system; TNM, tumor-node metastasis.

**Supplementary Table 2. Real time PCR primers used in this study**

| Genes | Forward | Reverse |
| --- | --- | --- |
| *lncAKHE* | 5’-CGTTACGGACAGTGTTTGCC-3’ | 5’-TGCATCCTTAGGTGATTTTGTTGT-3’ |
| *ACTB* | 5’-TCCATCATGAAGTGTGACGT-3’ | 5’-GAGCAATGATCTTGATCTTCAT-3’ |
| *HIF1a* | 5’-GAACGTCGAAAAGAAAAGTCTCG-3’ | 5’-CCTTATCAAGATGCGAACTCACA-3’ |
| *VEGF* | 5’-ATCACGAAGTGGTGAAGTTC-3’ | 5’-TGCTGTAGGAAGCTCATCTC-3’ |
| *MYC* | 5’-GGCTCCTGGCAAAAGGTCA-3’ | 5’-CTGCGTAGTTGTGCTGATGT-3’ |
| *TCF1* | 5’-CTGGCTTCTACTCCCTGACCT-3’ | 5’-ACCAGAACCTAGCATCAAGGA-3’ |
| *HES6* | 5’-AGCAGGAGCCTGACTCAGTT-3’ | 5’-AGCTCCTGAACCATCTGCTC-3’ |
| *HEY1* | 5’-GTTCGGCTCTAGGTTCCATGT-3’ | 5’-CGTCGGCGCTTCTCAATTATTC-3’ |
| *GLI1* | 5’-TGGATATGATGGTTGGCAAGTG-3’ | 5’-ACAGACTCAGGCTCAGGCTTCT-3’ |
| *GLI3* | 5’-GAAGTGCTCCACTCGAACAGA-3’ | 5’-GTGGCTGCATAGTGATTGCG-3’ |
| *YEATS4* | 5’-GCGGGAGAGTAAAGGGTGTT-3’ | 5’-ATGATACAGGGTTACCTCATTTCT-3’ |
| *NOTCH2* | 5’-CCAGGCTATCAGGGTGTCAA-3’ | 5’-ATGCCCTGGATGGAAAATGGAT-3’ |

**Supplementary Table 3. Primers used for *NOTCH2* promoter analysis in this study**

| Region | Forward | Reverse |
| --- | --- | --- |
| -2100~-1800 | 5’-CGGTGTTAAGATAAAGAAGCG-3’ | 5’-ACTTCTAATCATAATTCATCCT-3’ |
| -1800~-1500 | 5’-AGGCCTTGTATGTACCATGTT-3’ | 5’-GGATGTGGGAGTTTACAATCC-3’ |
| -1500~-1200 | 5’-GCTCCAGTTCACCTCTTGCTCC-3’ | 5’-GCAGCTACCTCTTAGGTCACAT-3’ |
| -1200~-900 | 5’-CTCTCCTGCCCTGGAAGTAAC-3’ | 5’-ATACTACACATTATCTGTGGAT-3’ |
| -900~-600 | 5’-CAAGGTTTTGCTATTACAGTGT-3’ | 5’-GAAAGCAGGATCTTGAAAGGTC-3’ |
| -600~-300 | 5’-CTACTCCAGAGCATTCCTCTA-3’ | 5’-TGCCGGGATCGTGAACTTGCAG-3’ |
| -300~0 | 5’-CTGATCGAGTTAAGGCGCGGC-3 | 5’-AGGGATCTACTACGAGTCACTG-3’ |
